# Expert consensus on optimal management of liver-related adverse events during CDK4/6 inhibitor therapy

**DOI:** 10.1007/s12094-026-04369-7

**Published:** 2026-05-10

**Authors:** Sonia Pernas, Xavier Forns, Olga Martínez-Sáez, Raúl Andrade, Alberto Amador, Elena López-Miranda, Begoña Bermejo, Beatriz Mateos, Monica Cejuela, María José Villanueva, Patricia Palacios Ozores, Mar Riveiro-Barciela, Meritxell Bellet

**Affiliations:** 1 Medical Oncology Department, Institut Catala d’Oncologia (ICO)-Institut de Recerca Biomèdica de Bellvitge (IDIBELL) L’Hospitalet, Barcelona, Spain; 2 Liver Unit, Hospital Clínic, IDIBAPS, Universitat de Barcelona, Barcelona, Spain; 3 Centro de Investigación Biomédica en Red de Enfermedades Hepáticas y Digestivas (CIBERehd), Instituto de Salud Carlos III, Madrid, Spain; 4 Medical Oncology Department, Hospital Clinic, Barcelona, Spain; 5 Translational Genomics and Targeted Therapies in Solid Tumors, IDIBAPS, Barcelona, Spain; 6 Department of Medicine, University of Barcelona, Barcelona, Spain; 7 Unidad de Gestión Clínica de Enfermedades Digestivas, Instituto de Investigación Biomédica de Málaga (IBIMA), Plataforma BIONAND, Hospital Universitario Virgen de La Victoria, Universidad de Málaga, Málaga, Spain; 8 Centro de Investigación Biomédica en Red de Enfermedades Hepáticas y Digestivas, CIBERehd, Madrid, Spain; 9 Gastroenterology Department, Hospital Universitari de Bellvitge, Hospitalet de Llobregat, Barcelona, Spain; 10 IDIBELL. Universitat de Barcelona, Barcelona, Spain; 11 Medical Oncology Department Hospital, Universitario Ramón y Cajal, Madrid, Spain; 12 Medical Oncology, Hospital Clínico Universitario de Valencia, Biomedical Research Institute INCLIVA, Valencia, Spain; 13 Medicine Department, Universidad de Valencia, Valencia, Spain; 14 Gastroenterology Department. Ramón, Cajal University Hospital, Madrid, Spain; 15 Medical Oncology Department, Hospital Virgen del Rocio (Seville, Spain), , Institute of Biomedicine of Seville (IBiS), Seville, Spain; 16 Medical Oncology. Medical Oncology Department, Complejo Hospitalario Universitario de Vigo, Galicia, Spain; 17 Medical Oncology Dpt. Breast and Gynecological Unit, Hospital Clínico Universitario de Santiago de Compostela, Galicia, Spain; 18 Instituto de Investigación Sanitaria de Santiago de Compostela (IDIS), Galicia, Spain; 19 Liver Unit, Internal Medicine Department, Hospital Universitari Vall d’Hebron, Vall d’Hebron Barcelona Hospital Campus, Barcelona, Spain; 20 Instituto de Salud Carlos III (Spain), European Reference Network on Hepatological Diseases (ERN RARE-LIVER), Madrid, Spain; 21 Breast Cancer Unit, Oncology Department, Hospital Universitari Vall d’Hebron and Vall d’Hebron Institute of Oncology (VHIO), Barcelona, Spain

**Keywords:** Liver enzyme alterations, CDK4/6 inhibitors, Drug-induced liver injury, Liver function monitoring

## Abstract

Hormone receptor-positive, human epidermal growth factor receptor 2-negative (HR + /HER2 −) breast cancer (BC) is the most frequently diagnosed subtype of BC, accounting for approximately 70% of cases. The introduction of cyclin-dependent kinase 4/6 inhibitors (CDK4/6i) in combination with endocrine therapy (ET) has significantly improved clinical outcomes and transformed the therapeutic landscape of HR + /HER2 − in both the metastatic and early settings.

Hepatotoxicity associated with CDK4/6i is increasingly recognized as a clinically relevant adverse event. Most cases are asymptomatic, occur early during treatment, and resolve with dose modification or management according to the Summary of Product Characteristics (SmPC). However, variability in monitoring and management persists in clinical practice. Key issues, such as corticosteroid use, treatment continuation and reintroduction of the CDK4/6i (either the same compound or another among the three globally approved agents) after liver toxicity recovery, remain insufficiently addressed by current evidence, despite the need for routine clinical decision-making.

To address these gaps, a Spanish multidisciplinary panel of hepatologists and medical oncologists developed expert consensus recommendations on liver toxicity in patients with HR + /HER2 − BC receiving CDK4/6i. This consensus provides practical guidance on the classification, monitoring and management of liver-related adverse events during CDK4/6i therapy and promotes proactive collaboration between oncology and hepatology teams to support treatment continuity and optimize patient outcomes.

## Introduction

Breast cancer (BC) remains the most commonly diagnosed malignancy and the leading cause of cancer-related mortality among women worldwide, with approximately 2.3 million new cases and 666,000 deaths reported in 2022 [1]. The predominant subtype is hormone receptor-positive, human epidermal growth factor receptor 2-negative (HR + /HER2 −), accounting for nearly 70% of all BC cases [2]. Endocrine therapy (ET) constitutes the backbone of systemic treatment for this population. In recent years, the addition of cyclin-dependent kinase 4/6 inhibitors (CDK4/6i) to ET has significantly reshaped the therapeutic landscape, becoming a key component of standard care in both metastatic and high-risk early-stage disease [3–5].

Among the three approved CDK4/6 inhibitors, ribociclib, palbociclib, and abemaciclib, the combination of these agents with ET has been established as a standard-of-care approach in first- and second-line treatment of HR + /HER2 − advanced breast cancer (ABC), demonstrating superior survival outcomes compared with ET alone [6, 7]. These advances in the metastatic setting led to the evaluation of CDK4/6i in early breast cancer (EBC). While the NATALEE and monarchE trials reported significant improvements in invasive disease-free survival (iDFS), with monarchE additionally showing an overall survival (OS) benefit, results have not been consistent across all agents. Specifically, palbociclib failed to demonstrate a significant improvement in iDFS when added to standard ET in the PALLAS and PENELOPE-B trials [8, 9].

Although CDK4/6i are effective, early treatment discontinuation due to adverse events (AEs) remains a concern, highlighting the need for proactive management to preserve efficacy, treatment adherence and quality of life. A systematic review of clinical trials and real-world data in ABC showed discontinuation rates due to AEs of 5.7% for ribociclib, 9.7% for palbociclib and 19.6% for abemaciclib, with grade (G) 3/4 toxicities reported in 73.6%, 58.4% and 79% of cases, respectively [10]. Neutropenia is the most frequent AE associated with palbociclib and ribociclib, while abemaciclib is more commonly linked to gastrointestinal toxicity, particularly diarrhea [11]. Liver enzyme elevations, such as alanine aminotransferase (ALT) and aspartate aminotransferase (AST), have been reported more frequently with ribociclib and abemaciclib [12, 13]. In the ribociclib trials, these AEs were classified as adverse events of special interest (AESI), leading to closer monitoring and reporting [3–5, 12–15].

Although typically transient and manageable, liver-related AEs can occasionally require dose adjustments, treatment interruptions and discontinuations. As such, clinical guidelines emphasize regular monitoring of liver function parameters to ensure timely management and maintain treatment continuity [16]. Liver AEs from CDK4/6i lack standard monitoring and management protocols in clinical practice. Interpreting liver enzyme increases during CDK4/6i therapy is challenging because of the limitations of the commonly used CTCAE criteria [17, 18]. CTCAE grading focuses primarily on ALT/AST levels, overlooking other liver function markers. In contrast, EASL DILI guidelines assess liver function more comprehensively by also including bilirubin and coagulation tests such as INR or prothrombin time. [19] This discrepancy may result in an overestimation of severity in asymptomatic patients with isolated liver enzyme elevations or, conversely, an underestimation of clinically significant injury when parameters indicative of liver function are impaired [19]. Alternative hepatology-focused tools such as the DILI Network severity score provide a more accurate evaluation of liver injury and may complement CTCAE in oncology settings [20, 21].

In response to these gaps, a national multidisciplinary consensus in Spain was developed to optimize the classification, monitoring and management of liver enzyme abnormalities in patients receiving CDK4/6i [20, 22].

The initiative promotes collaboration between oncologists and hepatologists to better manage liver-related AEs in HR + /HER2 − BC patients treated with CDK4/6i. The recommendations aim to improve treatment safety and patient outcomes through coordinated care.

## Methods

The literature search was performed using PubMed and other biomedical databases, employing Medical Subject Headings (MeSH) terms relevant to the topic. The search included the term [MeSH] and the following terms: “Breast Neoplasms”, “Cyclin-Dependent Kinase 4”, “Cyclin-Dependent Kinase 6”, “Protein Kinase Inhibitors”, “Alanine Transaminase/standards”, “Chemical and Drug induced liver injury/diagnosis”, “Drug-Induced Liver Injury”, “Liver Function Tests”, “Transaminases”, “Hypertransaminasemia”, “Adjuvant Chemotherapy” and “Estrogen Receptor-Positive Breast Cancer”.

The consensus on managing liver enzyme elevations during CDK4/6i therapy included hepatologists examining causes and detection, oncologists addressing clinical management, and a joint session merging insights. The outcome was guidelines for identifying, monitoring, and treating patients taking CDK4/6i.

The principal consensus statements emerging from the Panel Discussion are presented below.

## Drug-Induced liver injury (DILI) associated with CDK4/6i: diagnosis and risk factors

### Diagnosis and mechanism of action of DILI

Drug-induced liver injury (DILI) is defined as an adverse reaction to drugs or other xenobiotics that may occur predictably with toxic doses or unpredictably with commonly used medications, leading to liver injury involving hepatocytes and other liver cells [23]. Diagnosing DILI is challenging because it requires ruling out other causes of liver injury. Key factors include timing, improvement after discontinuing the drug, recurrence with re-exposure, the drug’s known hepatotoxicity, and clinical presentation. There are no specific DILI biomarkers; tests like biopsy, imaging, and serologic markers mainly help exclude alternative diagnoses [19].

Determining the true incidence of DILI remains difficult. The range of drugs associated with liver injury is continually expanding [22], encompassing not only commonly prescribed agents but also specialized treatments like certain oncology and neurology drugs [19].

The most challenging form of DILI is the idiosyncratic type, due to its unpredictable nature. It typically does not correlate with dosage, affects only a small fraction of individuals exposed and has variable onset times with delayed latency in different cases. This form presents multiple phenotypes, complicating recognition and differential diagnosis [19].

CDK4/6i exhibit distinct chemical and pharmacokinetic profiles, including CYP3A4-mediated metabolism and biliary excretion as their principal elimination route [24]. However, the precise mechanism of CDK4/6i-induced liver injury is not fully understood, and both the natural history of this toxicity and its optimal management are still poorly described [18]. The phase 2 randomized AMALEE trial, which compared ribociclib doses of 400 mg and 600 mg [18]. The phase 2 AMALEE trial found similar rates of liver function abnormalities in patients taking ribociclib at 400 or 600 mg, indicating hepatic toxicity is not likely dose-dependent. Pharmacogenetic analyses point to intracellular pathways, such as those involving EP300, SIRT1, STAT3 and HSP90AA1, as contributors to liver injury with CDK4/6i, highlighting complex biological mechanisms beyond simple toxicity or pharmacokinetics [25]. Additionally, clinical observations indicate a potential inflammatory component without clear evidence of an immune-mediated mechanism, as well as a possible association between hepatic and biliary alterations and other factors, like hepatic transporters [25].

**Fig. 1:**
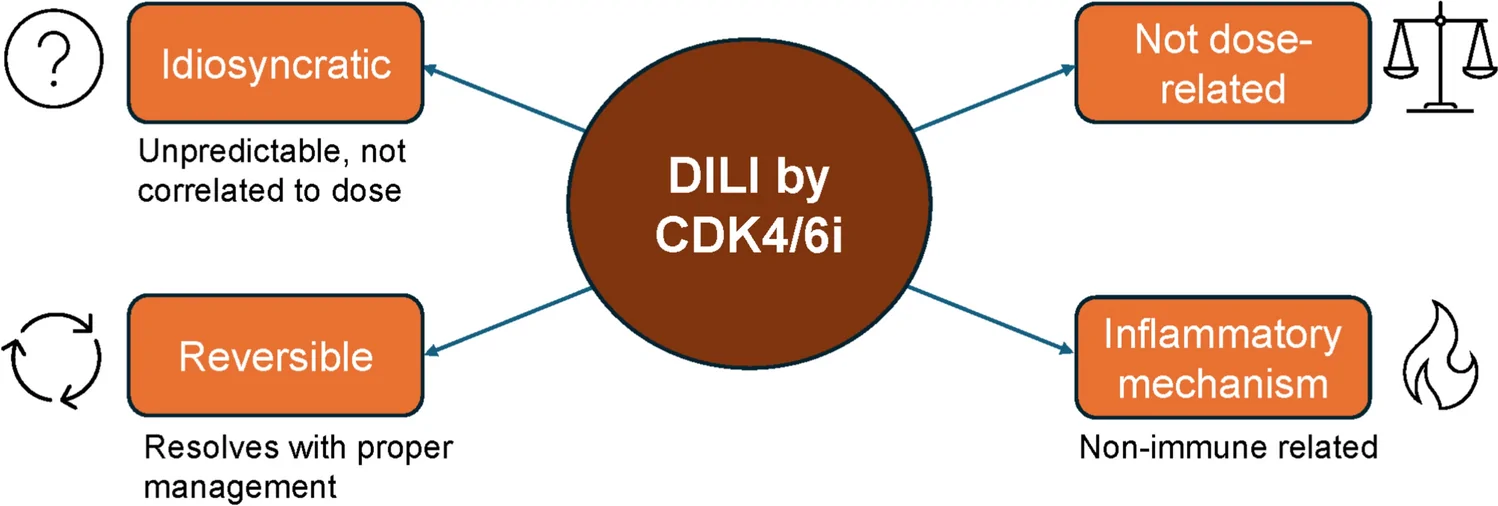
Key features of CDK4/6i DILI

In conclusion, expert consensus suggests that DILI induced by CDK4/6i therapy follows an idiosyncratic and largely reversible pattern, with no clear evidence of dose dependence (Fig. 1) [18]. Most cases lack autoimmune hepatitis markers (auto-antibodies, elevated IgG), and positive responses to corticosteroids suggest an inflammatory rather than immune-mediated mechanism [26]. While ribociclib and abemaciclib inhibit hepatic transporters like BSEP; this does not fully explain liver changes seen clinically. The complexity of liver injury highlights the need for individualized monitoring and management [20, 24, 27, 28].

### Risk factors

Factors like age, race, BMI, fatty liver disease, alcohol use, menopausal status, prior treatments, and concurrent medications may affect DILI risk. Although no single factor clearly increases risk for patients taking CDK4/6i, evidence suggests these variables can interact and should be considered during clinical monitoring [29, 30]. Older age did not consistently increase liver toxicity risk. Harbeck et al.’s pooled analysis of 6836 ribociclib-treated patients from multiple studies found no consistent age-related rise in hepatotoxicity. However, as the study was descriptive, no formal statistical analysis was conducted for predictive variables [31]. An elevated risk of DILI with abemaciclib has been suggested among Asian patients. In the Japanese subpopulation, higher proportions of ALT and AST elevations were reported with abemaciclib in the MONARCH 2 and 3 trials, with rates of 30–35% and 50%, respectively [32].

A preplanned MONALEESA 7 trial analysis found no increased liver toxicity among Asian women compared to non-Asians. However, since the study focused on Korean and Asian patients without separate data for Japanese patients, this may account for inconsistencies in DILI risk across studies [33].

Several studies have shown that high BMI (> 30) and increased abdominal circumference are strongly associated with hepatic steatosis and, consequently, with a higher risk of DILI [34, 35]. Furthermore, dyslipidemia parameters, such as low HDL-cholesterol and hypertriglyceridemia, appear to further increase this susceptibility [34, 36]. Real-world data provides additional insights into the contribution of BMI and hepatic steatosis to DILI susceptibility. In a Spanish retrospective series, Mascaró et al. observed that 59% of patients who experienced DILI induced by CDK4/6i (26 cases) had a BMI > 25 [37]. A Spanish study found that 31% of DILI patients had fatty liver on baseline CT. Baseline hepatic steatosis with any ALT elevation was marginally linked to DILI compared to controls (*p* = 0.04) [32, 38]. In addition, a retrospective Japanese study of 52 patients treated with abemaciclib reported that 29% (*n* = 15) experienced new liver enzyme elevations, mostly G1 or 2 and identified liver steatosis as an independent risk factor for these elevations (*p* = 0.047). The pooled analysis of pivotal ribociclib trials could not address this issue, as information on the presence of hepatic steatosis was not prospectively collected [31]. These gaps in knowledge underscore the need for further research into the interplay between BMI, hepatic steatosis and other metabolic determinants of susceptibility to DILI.

Predictive factors for DILI increase metabolic stress in the liver. Although data are scarce, hormonal shifts and polypharmacy contribute to higher DILI risk. The Hepatic Steatosis Index, factoring ALT/AST ratio, BMI, sex, and diabetes, reliably predicts Metabolic Dysfunction-Associated Steatotic Liver Disease, underscoring the connection between metabolism and liver health. [39, 40]. While alcohol consumption and DM have been proposed as potential risk factors among the experts, their association with DILI has not been clearly established. [34, 35]. Collectively, these observations highlight the need for a nuanced and individualized approach to monitoring and mitigating additional hepatotoxicity risk factors in patients receiving CDK4/6i.

Regarding baseline ALT/AST elevations per se, irrespective of their cause, the ribociclib pooled analysis reported a numerically higher proportion of on-treatment AST or ALT elevations among patients with mild baseline liver impairment [31]. Therefore, baseline abnormalities cannot be ruled out as potential predictive factors for ribociclib-related DILI.

The influence of menopausal status on the risk of CDK4/6i-associated liver injury remains uncertain and is currently a subject of debate. In the case–control study by Cano-Vega et al., premenopausal status combined with CT-detected hepatic steatosis was associated with an odds ratio (OR) of 8.6 (95% CI 2.1–43.6, *p* < 0.004) for developing G ≥ 2 transaminases elevation with any of the three approved CDK4/6i [38]. The authors suggested that the rapid metabolic changes induced by ovarian function suppression might synergize with CDK4/6i to induce DILI in patients with underlying steatosis. However, the pooled analysis of pivotal trials did not support that hypothesis, with the postmenopausal patients or those not receiving goserelin being the ones with a higher rate of AST/ALT rises on treatment [38]. Moreover, in the MONALEESA-7 trial, which exclusively enrolled premenopausal patients treated with goserelin, the incidence of liver injury was numerically lower than that reported in MONALEESA-2, which included only postmenopausal patients (ALT/AST elevation ≥ G3: 5%/4% vs 9%/5%, respectively) [41]. These conflicting results highlight the need for larger studies to determine if menopausal status affects liver safety during CDK4/6i treatment. Future research should report recent bilateral oophorectomy, which removes the need for GnRH analogs, instead of relying solely on categorical definitions of menopausal status.

Harbeck et al.’s pooled analysis and a Spanish case–control series found no difference in DILI risk between aromatase inhibitors (AIs) and fulvestrant. In contrast, Japanese studies showed that AI use, compared to fulvestrant, was the strongest predictor of abemaciclib-related liver toxicity (*p* = 0.005) [32]. Previous studies suggest AI with tegafur-uracil may raise liver toxicity risk, likely due to inflammation from estrogen deprivation combined with abemaciclib’s immune effects. More information about AI types used in large trials like monarchE and NATALEE would help clarify hepatotoxicity risks across endocrine therapies.

The development of liver enzyme abnormalities during CDK4/6i therapy does not appear to be influenced by the presence of liver metastases or prior chemotherapy. In the case series by Meunier et al., liver metastases were present in only 5 out of 22 patients who experienced CDK4/6-related hepatitis, suggesting that hepatic tumor burden is not a key driver of toxicity [18]. This observation is consistent with findings from other real-world studies. [26, 38] Similarly, liver metastases were not identified as a potential risk factor in the pooled analysis by Harbeck et al. Furthermore, the AMALEE trial reported similar rates of hepatic alterations at both 400 and 600 mg ribociclib doses, regardless of liver metastasis status or baseline liver function [42]. Moreover, in most reported cases, imaging studies explicitly ruled out tumor progression as the cause of liver enzyme elevations [43, 44].

Taken together, these findings outline potential risk profiles for liver enzyme elevations during CDK4/6i therapy. Importantly, while factors such as hepatic steatosis, menopausal status or endocrine partner may influence individual susceptibility, their identification should not preclude treatment but rather prompt closer monitoring and proactive management of modifiable comorbidities to ensure patient safety [29, 30].

## Liver enzymes elevation classification

DILI can range from mild, asymptomatic cases to rare, fatal outcomes, making objective classification difficult. A standardized system is important for managing patients with DILI, including those on CDK4/6i. The Common Terminology Criteria for Adverse Events (CTCAE) v5.0, commonly used in oncology, classifies drug-induced adverse events according to severity from mild (G1) to fatal (G5), based on clinical, radiological, or lab findings [17]. As previously mentioned, transaminase levels alone are insufficient to determine the severity of liver injury and, therefore, bilirubin and coagulation parameters should always be evaluated in such cases [19].

The term DILI refers to liver injury caused by pharmaceutical agents, herbal products, or other xenobiotics. It encompasses a wide spectrum of liver abnormalities, ranging from mild and transient elevations in liver enzymes to acute liver failure [23]. Idiosyncratic DILI is best described as an adverse hepatic reaction that is unexpected based on the drug’s pharmacological action and is distinct from dose-dependent hepatotoxicity such as that seen in drug overdose.

Unlike the CTCAE, which evaluates liver-related parameters individually, DILI criteria and degree of severity have been established by an international DILI Expert Working Group to define standardized thresholds for categorizing patterns of liver injury, as well as their severity and chronicity. These criteria consider the degree of elevation in alanine aminotransferase (ALT), alkaline phosphatase (ALP) and bilirubin, together with the International Normalized Ratio (INR) and the presence or absence of clinical symptoms. DILI definitions and categories are summarized in Table 1 [21].

**Table 1:** DILI severity index: adapted from Aithal et al.

Category	Severity	Description
1	Mild	Elevated ALT/ALP concentration reaching criteria for DILI* but bilirubin concentration < 2 × ULN
2	Moderate	Elevated ALT/ALP concentration reaching criteria for DILI* and bilirubin concentration ≥ 2 × ULN or symptomatic hepatitis
3	Severe	Elevated ALT/ALP concentration reaching criteria for DILI*, bilirubin concentration ≥ 2 × ULN and one of the following: • International normalized ratio (INR) ≥ 1.5 • Ascites and/or encephalopathy, disease duration < 26 weeks and absence of underlying cirrhosis • Other organ failure is considered to be due to DILI [18]
4	Fatal or transplantation	Death or transplantation due to DILI*

*Any one of the following: a) More than or equal to fivefold elevation above the ULN for ALT; b) More than or equal to twofold elevation above the ULN for ALP (particularly with accompanying elevations in concentrations of 5′-nucleotidase or γ-glutamyl transpeptidase (GGT) in the absence of known bone pathology driving the rise in ALP level); c) More than or equal to threefold elevation in ALT concentration and simultaneous elevation of bilirubin concentration exceeding 2 × ULN. Level of evidence: 2b (exploratory/retrospective cohort studies)

Additionally, Hy’s law criteria identify a high-risk pattern of liver injury characterized by ALT or AST ≥ 3 × the ULN, total bilirubin > 2 × ULN and ALP ≤ 2 × ULN, in the absence of alternative etiologies. The occurrence of two or more such cases during drug clinical development is considered predictive of a significant risk of serious liver injury [45].

In summary, although CTCAE v5.0 remains a valuable tool, integrating additional biochemical parameters such as total bilirubin (within DILI-specific frameworks) offers a more comprehensive and relevant assessment of liver function and severity of liver injury.

Finally, it is worth noting that the enzymatic pattern of CDK4/6i-associated DILI is predominantly hepatocellular, characterized by elevations in AST and ALT; however, it may also present as a predominantly cholestatic pattern (with increased ALP or GGT) or as a mixed pattern [26]. The pattern of DILI indicates the type and prognosis of liver injury rather than the specific drug involved. Hepatocellular injury poses a greater risk of acute liver failure, cholestatic injury usually has a longer, less severe course, and mixed injury falls between these outcomes. The prognostic significance of these DILI patterns for CDK4/6i remains unstudied [19, 46].

## Incidence of liver-related AEs associated with CDK4/6i and potential prognosis impact

### Liver enzyme elevations in the main clinical trials

Across pivotal trials investigating CDK4/6i, reported elevations in liver enzymes were frequent laboratory abnormalities (Table 2) [3, 5, 47–50]. In the MONALEESA, MONARCH and PALOMA programs, the incidence of G3/4 ALT elevations ranged between 5 and 9% for ribociclib [5, 51], around 4–7% for abemaciclib, [4] and notably lower for palbociclib [3]. The recently published NATALEE and monarchE studies in the adjuvant setting also reported similar elevations in serum transaminases. [12, 13] Additional data from a pooled analysis by Harbeck et al. of the MONALEESA-2, -3, -7, CompLEEment-1 and NATALEE studies further support these findings, with a total incidence of transaminase elevations of any grade of 18.4% and G3/4 elevations of < 9% across all trials [31, 52]. However, in the recently presented six-month neoadjuvant cohort of the RIBOLARIS trial, any-grade and G3/4 transaminase elevations were observed in 22% and 12% of patients, respectively, which were slightly higher than previously reported [53]. Except for this last trial, the median time to onset of G ≥ 3 transaminase elevations ranged from 2.6 to 4.9 months across the remaining trials, suggesting an early, time-dependent susceptibility window.

**Table 2:** Comparative incidence of liver injuries with CDK4/6i from pivotal phase III trials in ABC and EBC. Data were retrieved from the following references [3, 13, 14, 33, 41, 48–51, 53, 55–57]

	Ribociclib					Abemaciclib			Palbociclib	
	MONALEESA-2	MONALEESA-3	MONALEESA-7	NATALEE	RIBOLARIS	MONARCH 2	MONARCH 3	monarchE	PALOMA-2	PALOMA-3
Setting	ABC Postmenopausal patients	ABC Postmenopausal patients	ABC Premenopausal patients	EBC Pre and postmenopausal patients	EBC Pre and postmenopausal patients	ABC Pre and postmenopausal patients	ABC Postmenopausal patients	EBC Pre and postmenopausal patients	ABC Postmenopausal patients	ABC Pre and postmenopausal patients
Intervention	Ribociclib plus letrozole/letrozole	Ribociclib plus fulvestrant/placebo plus fulvestrant	Ribociclib plus goserelin plus ET (tamoxifen or letrozole or anastrozole)/placebo plus goserelin plus ET	Ribociclib plus nonsteroidal aromatase inhibitor (NSAI)	Ribociclib plus ET vs standard chemotherapy plus ribociclib plus ET	Abemaciclib plus fulvestrant/fulvestrant	Abemaciclib plus NSAI/placebo plus NSAI	Abemaciclib plus ET	Palbociclib plus letrozole/placebo plus letrozole	Palbociclib plus fulvestrant/placebo plus fulvestrant
N patients (total)	668	726	672	5101	1100	669	493	5,637	666	521
N patients treated with CDK4/6i	334	484	335	2549	1100	446	328	2808	444	347
Increased ALT G1/2**, % (number of cases)	6.2% (21) vs 4.8% (16)	6% (29) vs NR	7.4% (25) vs NR	11.8% (300) vs 4.9% (119)	G1/2 10.2% (70)	9.3% (41) vs NR	11% (36) vs NR	NR	NR	NR
Increased AST G1/2**, % (number of cases)	9.2% (31) vs 4.8% (16)	7.2% (35) vs NR	8.4% (28) vs NR	12.1% (308) vs 5.2% (126)		9.9% (44) vs NR	13.1% (43) vs NR	NR	NR	NR
Increased ALT G3/4**, % (number of cases)	9.3% (31) vs 1.2% (4)	8.5% (41) vs 0.4% (1)	5% (17) vs 1% (3)	7.3% (181) vs 0.75% (16)	12% G3/4 transaminase elevation	4.1% (18) vs 1.8% (4)	7.0% (22) vs 1.9% (6)	1.6% (46) vs 0.5% (13)	1 had G4 event	1.7% (6) vs 0
Increased AST G3/4**, % (number of cases)	5.7% (19) vs 1.2% (4)	6.0% (29) vs 0.8% (2)	4.8% (13) vs 1% (3)	4.4% (112) vs 0.5% (12)		2.3% (10) vs 2.7% (6)	3.8% (12) vs 0.6% (1)	2.3% (64) vs 0.5% (16)	No cases reported	3.2% (11) vs 2.3% (4)^$^
Hy’s Law/liver failure	Four cases of Hy’s law*	Two cases of Hy’s law	No cases reported	Eight cases of Hy’s law	No cases reported	No cases reported	No cases reported	No cases reported	No cases reported	2 patients with liver failure

NR: not reported

*Three were suspected to be drug-related; two cases had features of autoimmune hepatitis; none of these cases resulted in death and the aminotransferase and bilirubin levels returned to normal in all four patients within 98–154 days after the discontinuation of ribociclib

**Experimental vs control arm

Importantly, the median duration of G ≥ 3 events in the MONALEESA trials, NATALEE and CompLEEment-1 ranged from 0.7 to 1.6 months (the range spans from 0.5 in MONALEESA-3 to 2.0 in CompLEEment-1), and these abnormalities generally resolved following temporary dose interruptions and/or dose reductions. Additionally, based on data from phase 3 trials of ribociclib in the first-line advanced setting, mild baseline AST elevation did not increase liver injury risk. [30] Most affected patients, particularly those with G1 liver toxicity, continued ribociclib for over two years, indicating long-term treatment is feasible with proper monitoring [54].

For palbociclib, pivotal trials consistently confirmed a low incidence of severe liver enzyme elevations. In PALOMA-2, only one case of G4 ALT elevation was reported, with no G3/4 AST elevations [3]. In PALOMA-3, G3/4 ALT and AST elevations occurred in 2.3% and 3.2% of patients, respectively [50].

In MONARCH 2, G3/4 ALT and AST elevations were reported in 4.1% and 2.3% of patients, respectively [49], whereas in MONARCH 3, the incidences were higher, at 7.0% and 3.8%, respectively [48]. In the adjuvant setting, the monarchE trial reported G3/4 ALT elevations in 2.3% and AST elevations in 1.6% of patients [12].

As previously mentioned, isolated evaluation of serum liver enzymes could overestimate the severity of DILI, which is more accurately determined by measuring bilirubin levels (Table 1) [21]. Therefore, assessing the incidence of bilirubin elevations reported across pivotal CDK4/6i trials is essential for a more accurate characterization of hepatotoxicity.

In the MONALEESA trials, Hy’s law criteria were met by 0.52% of patients. Harbeck et al.’s pooled analysis found only 21 of 6869 patients (0.3%) qualified [51]. In the PALOMA-3 trial, two patients presented liver failure with palbociclib [50], whereas no Hy’s Law cases were reported with abemaciclib in MONARCH 2 or MONARCH 3 studies [48, 49].

These findings highlight that bilirubin elevations were infrequent across pivotal CDK4/6i studies. Consequently, only a small subset of liver-related AEs would fulfill the criteria for moderate or severe DILI. Hence, although liver enzyme elevations are categorized as G3 or 4 by CTCAE standards, applying DILI criteria would likely result in a more nuanced and clinically meaningful interpretation of hepatic AEs (Table 2).

#### *$ based on the overall survival analysis *[50]

### Liver-enzyme elevations with CDK4/6i: pharmacovigilance and real-world data

Although CDK4/6i rarely cause DILI, their potential severity and impact on treatment are important. FAERS data suggest ribociclib and abemaciclib have more liver-related side effects than palbociclib, but this may be due to reporting bias and limited clinical detail, especially about lab results like bilirubin or INR [25].

Findings from real-world evidence (RWE) studies align with these safety signals and provide additional insights into palbociclib or abemaciclib associated liver toxicity, which was less extensively characterized in pivotal trials compared with ribociclib. Consistent with previous phase 3 studies, multiple retrospective cohorts have reported that liver enzyme elevations G ≥ 2 appear more frequently with ribociclib and abemaciclib than with palbociclib (Table 3). Most transaminase elevation events occurred early during treatment and were reversible, either spontaneously or following dose adjustments. In selected cases, corticosteroids were used and some patients were successfully rechallenged with an alternative CDK4/6i. Although severe cases (G3–4) were rare, they led to treatment discontinuation [18, 26, 38, 58].

**Table 3:** Summary of the main RWE series addressing liver enzyme alterations with ET combined with CDK4/6i

Study	Population	Design	Liver enzymes elevations
She et al., 2023 [25]	84,462 AE reports for any CDK4/6i	Pharmacovigilance (FAERS database)	Reporting odds ratio of disproportionality analyses for CDK4/6i-related DILIs Palbociclib (*n* = 2256): 0.70 (95% CI 0.67, 0.73*)* Abemaciclib (*n* = 587): 2.37 (95% CI 2.18, 2.58) Ribociclib (*n* = 1949): 2.60 (95% CI 2.48, 2.72)
Rugo et al., 2022 [58]	958 patients with ABC treated with ribociclib	Retrospective, real-world data	G ≥ 2 ALT/AST in 70 patients (7%) In the patients with resolution of ALT/AST elevation to G1 or 0, resolution occurred at a median of 14.5 days (range, 6–88 days) after first G ≥ 2 elevation
Meunier et al., 2021 [18]	22 patients with G3—4 CDK4/6i-ILI	Retrospective case series	Severity of induced liver injury: Mild (82%) Moderate (14%)
Vega-Cano et al., 2023 [38]	472 patients treated with CDK4/6i: 26 cases with AST and/or ALT elevations G2 or higher	Retrospective, 1 center	G ≥ 2 ALT/AST in 26 patients (5.5%) Palbociclib (*n* = 8): 7 (87%) G3 Abemaciclib (*n* = 9): 4 (44%) G3, 1 (12%) G4 Ribociclib (*n* = 9): 2 (23%) G3, 1 (11%) G4
Riveiro-Barciela et al. 2025 [26]	1716 patients treated with CDK4/6i: 85 patients with AST and/or ALT elevation G2 or higher	Retrospective, multicenter study	G ≥ 2 ALT/AST in 85 patients (4.9%) Palbociclib: 14/861 (1.6%) Abemaciclib: 28/351 (8.0%) Ribociclib: 43/504 (8.5%)
Bas et al. 2024 [59]	24 patients with ribociclib-ILI	Retrospective, multicenter study	54/845 liver toxicity (G1/2) 24/845 liver toxicity (≥ G3) Dose reduction (G3): 11/845 (1.3%) 5 discontinuations (G4). One successful rechallenge to ribociclib

**Fig. 2:**
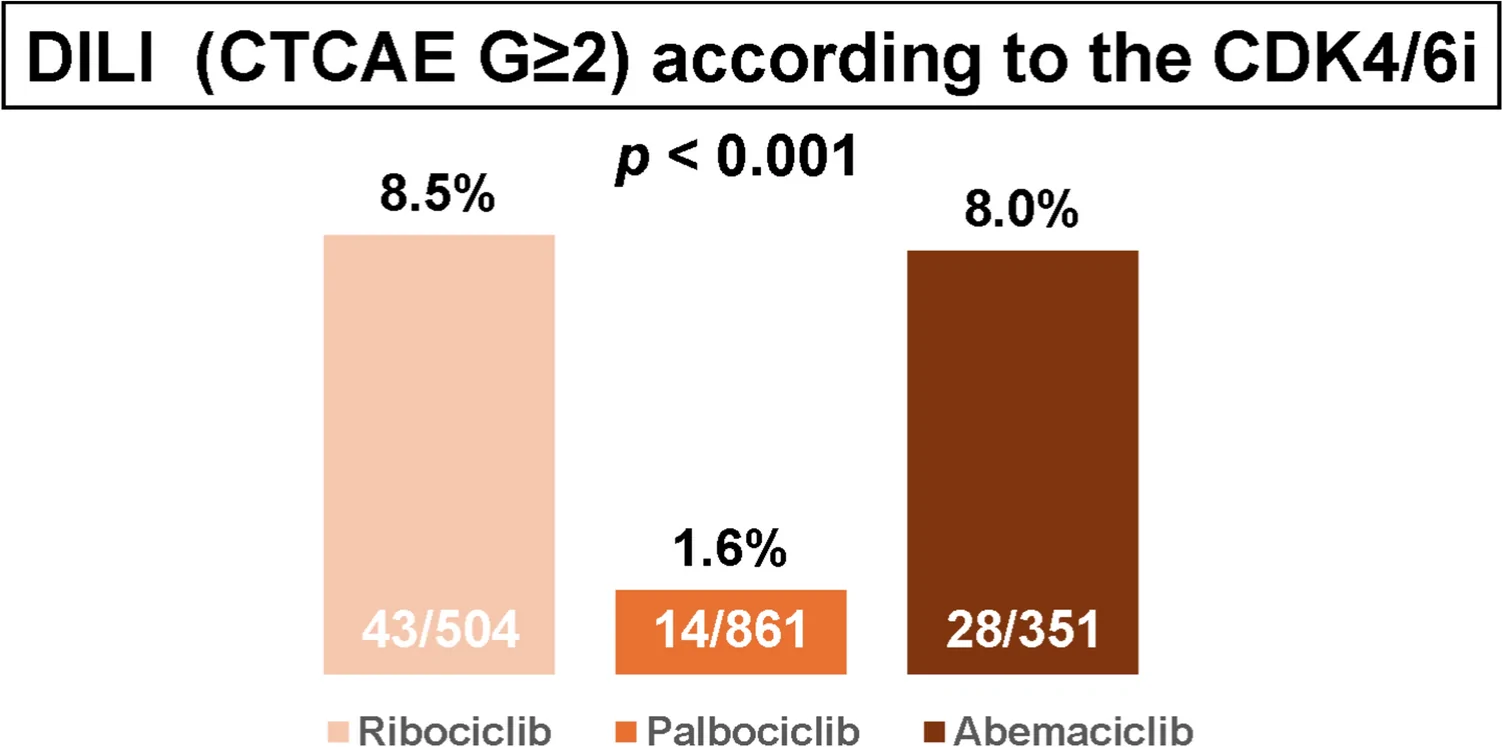
DILI (CTCAE ≥ G2) with ribociclib, palbociclib and abemaciclib in a multicenter retrospective Spanish trial [26]

Data from a retrospective study conducted in Vall d’Hebron Hospital (Barcelona, Spain) evaluated liver enzyme elevations in 472 patients receiving first-line CDK4/6i treatment for ABC. Transaminases increase of G ≥ 2 was observed in 26 patients (5.5%) [38]. Another Spanish multicenter retrospective study identified DILI induced by CDK4/6i CTCAE G ≥ 2 in 4.9% of 1716 patients treated with CDK4/6i (Fig. 2). Most cases were G3 or 4 (68%) and occurred within the first three months of treatment.

CDK4/6i-associated DILI usually shows a hepatocellular pattern (most common among patients treated with ribociclib and palbociclib) but can also appear as cholestatic (in patients on abemaciclib) or mixed [26]. The pattern of DILI provides insights into the type of liver injury and its prognosis. Hepatocellular injury, when associated with bilirubin increase, carries a higher risk of acute liver failure, while cholestatic injury typically requires a more prolonged course to ALT normalization and lower risk of severe liver outcomes. However, it should be stressed that the prognostic implications of these different DILI patterns in the context of CDK4/6i have not yet been studied.

The prognostic development of DILI induced by CDK4/6i has been analyzed in 2 retrospective series with 26 patients (transaminases elevation ≥ G2 with any CDK4/6i) and 24 patients (transaminases elevation ≥ G3 with ribociclib), respectively [38, 59]. Importantly, although limited by small sample size and the retrospective nature of the two series, both studies consistently describe a statistically significant negative impact on progression-free survival (PFS) in patients who develop DILI compared with a control group without liver toxicity.

## Liver function assessment, monitoring and management during CDK4/6i therapy

### Baseline evaluation

Before initiating treatment with CDK4/6i, baseline liver function assessment is recommended in clinical practice and should include measurements of ALT, AST and total bilirubin, as well as an initial evaluation of liver risk factors, including hepatic steatosis, alcohol consumption and concomitant use of potentially hepatotoxic medications. [28, 60] Although baseline liver enzyme elevations do not seem to be associated with an increased risk of treatment-related hepatotoxicity, particularly with ribociclib [30], patients presenting risk factors for DILI may benefit from a more structured baseline evaluation.

### Review of concomitant medication

A comprehensive review of concomitant medications is essential before and during treatment with CDK4/6i, as they may act as confounding factors in the assessment of liver injury [61]. This is particularly relevant in older patients, who often have multiple comorbidities and chronic treatments [62, 63]. A detailed medication history may help identify potential hepatotoxic agents and improve a more accurate evaluation of liver-related AEs [61].

### Liver function monitoring during CDK4/6i therapy

Monitoring liver function in patients treated with CDK4/6i is advisable to ensure treatment safety and optimize clinical outcomes. According to the summary of product characteristics (SmPC) for ribociclib, palbociclib and abemaciclib, baseline and periodic liver function testing, including ALT, AST, total bilirubin and INR, is recommended [27, 28, 60].

Routine monitoring includes measuring ALT, AST and bilirubin levels every two weeks during the first two cycles, at the beginning of cycles 3 through 6 and subsequently as clinically indicated [64]. Including INR and ALP might enhance early detection of functional liver injury, and may facilitate the identification of liver abnormalities, especially in patients with pre-existing liver dysfunction or relevant comorbidities [31].

As previously noted, the DILI International Expert Working Group has established standardized diagnostic criteria incorporating ALT, ALP, total bilirubin and PT/INR to categorize hepatocellular, cholestatic and mixed injury patterns, and to assess severity. This comprehensive evaluation was included in the pivotal trials and in all the ribociclib protocols. However it is not used in standard oncology clinical practice. Their inclusion could facilitate earlier detection of clinically relevant liver injury, especially in patients with metabolic comorbidities or other risk factors [25].

### Algorithm for monitoring and management of liver enzyme elevations during CDK4/6i therapy. Evidence of rechallenge and switching of CDK4/6i

**Fig. 3:**
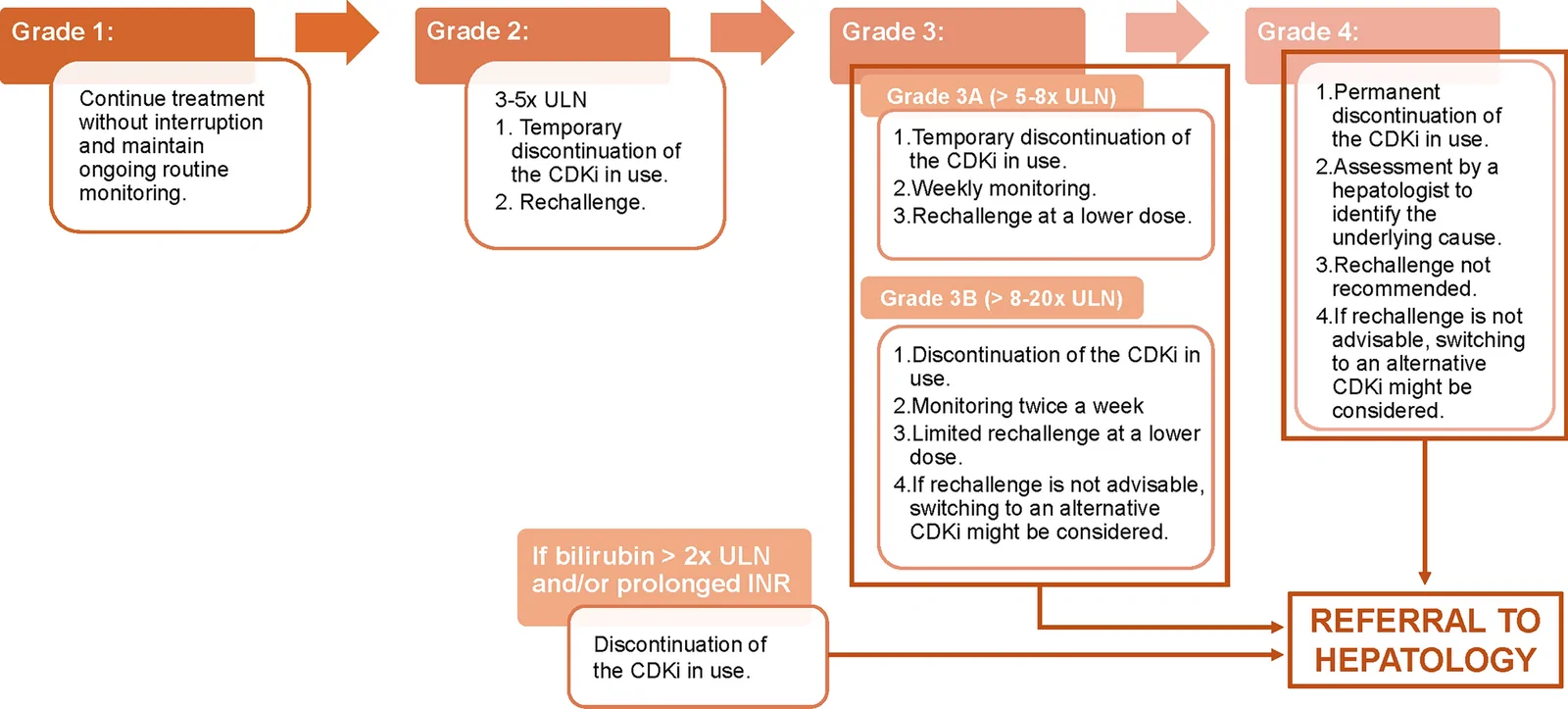
Monitoring protocol during treatment with CDK4/6i, in both advanced and adjuvant settings. Figure created from the expert consensus of the co-authors. *Consider corticoid treatment in G3 without improvement after 2–4 weeks, or if BT ≥  × 2 ULN, dose: 0.5–1 mg/kg/d

The management of liver enzyme elevations in patients receiving CDK4/6i requires a structured approach, considering both the degree of enzyme elevations and the presence of clinical and/or analytical functional liver impairment [17, 21]. Accordingly, we propose an adapted monitoring algorithm that extends beyond the recommendations provided in the Summary of Product Characteristics (SmPC) for ribociclib, with the aim of guiding clinical decision-making based on liver enzyme levels, bilirubin status and INR values (Fig. 3). In contrast, the SmPCs for palbociclib and abemaciclib do not provide specific guidance regarding rechallenge in the setting of liver enzyme elevations. In this algorithm, discontinuation due to liver enzyme elevations refers to a temporary pause of CDK4/6i treatment. If recovery occurs, a rechallenge, defined here as reintroducing the same agent, may be attempted.

For patients with G1 liver enzyme elevations according to CTCAE v5.0 (ALT/AST up to 3 × ULN) treatment should be continued without discontinuation, with ongoing routine monitoring.

In case of G2 elevations (ALT/AST 3–5 × ULN), temporary treatment discontinuation is indicated [29]. Rechallenge at the same dose is appropriate once ALT/AST levels decrease to G1 or baseline level, following the approach used in the NATALEE trial [52]. However, according to the SmPC of ribociclib, rechallenge is formally recommended only after full normalization of ALT/AST to baseline values. In the event of recurrent G2 elevation, ribociclib should be resumed only at the next lower dose level [27].

For G3 elevations (ALT/AST > 5–20 × ULN)**,** given the wide range of values in this category, a redefined distinction is proposed by the hepatologist panelists. These two subcategories are based on standard clinical management for DILIs induced by multiple non-oncologic drugs and consider ULN values. The differences between A and B are subtle; however, this differentiation could improve management.

- G3a (> 5–8 × ULN): temporary discontinuation. Reintroduction may be considered once ALT/AST levels improve to G1, with weekly close monitoring including bilirubin levels and INR. Rechallenge is only allowed when bilirubin levels have not gone above 2 × ULN.
- G3b (> 8–20 × ULN): discontinuation of the CDK4/6i in use. Twice-a-week monitoring and hepatology referrals are advised. Rechallenge should be limited and considered with caution based on clinical circumstances; CDK4/6i may be restarted at a reduced dose only if bilirubin has not exceeded 2 × ULN.

For G4 elevations (ALT/AST > 20 × ULN or with significant liver dysfunction), permanent discontinuation of the CDK4/6i is recommended. Rechallenge is contraindicated also in these cases [27, 28, 60].

Any elevation above 2 × ULN in bilirubin or prolonged INR, irrespective of transaminase level, warrants hepatology consultation to ensure accurate assessment and management. Permanent discontinuation of the CDK4/6i is recommended and a rechallenge with the same agent is contraindicated in these cases [27, 28, 60].

Unlike rechallenge, switching to a different CDK4/6i after recovery has not been evaluated in the pivotal clinical trials and, therefore, should be interpreted with caution. Real-world data from Riveiro-Barciela et al. indicate that CDK4/6i reintroduction (either with the same agent or with other approved CDK4/6i) was attempted in 70 patients (82%). Recurrent DILI occurred in 19 cases (27%) and all events were classified as mild according to the DILI severity index. The rate of CDK4/6i reintroduction was significantly lower among patients with criteria of moderate DILI (*p* = 0.022). However, CDK4/6i reintroduction rates did not differ according to CTCAE grade (*p* = 0.507) or the initially administered CDK4/6i (*p* = 0.283). The risk of recurrence was not significantly associated with the specific CDK4/6i initially administered (*p* = 0.773). Notably, among the 38 patients who developed ribociclib-related DILI, recurrence rates were lower when a switching strategy was implemented (*p* = 0.006). No DILI recurrences were observed among those switched to palbociclib (0/12), whereas recurrence occurred in 1/8 patients (13%) switched to abemaciclib and in 9/18 patients (50%) rechallenged with ribociclib. [26].

The consideration of corticosteroid therapy for managing persistent ALT/AST elevations is supported by evidence of an underlying inflammatory component in CDK4/6i-induced liver injury. Two retrospective series reported that 40.9% (9/22) and 19% (16/85) of patients received corticosteroids for CDK4/6i-related hepatotoxicity [18, 26]. In both studies, biochemical features suggestive of more severe liver injury were observed. In one study, recovery time was longer among patients treated with corticosteroids, although this does not exclude the possibility that the delay was related to more severe underlying hepatic injury in this subgroup. Due to the lack of clear evidence, the panel agreed that decisions should be tailored to individual clinical circumstances. Corticosteroid treatment could be discussed when liver injury is not resolved promptly after discontinuation of the CDK4/6i (e.g., after approximately 2 weeks) and after ruling out an alternative diagnosis. If bilirubin levels exceed 2 × ULN, prompt referral to a hepatologist is encouraged to evaluate the initiation of corticosteroid therapy.

Importantly, no firm consensus has been established regarding whether the ET partner should be concomitantly interrupted in cases of significant hepatic alterations, as the available evidence remains limited [47]. Treatment decisions should be individualized, weighing the risks of cancer progression against the severity of liver injury.

## Conclusions

CDK4/6 inhibitors have significantly improved outcomes for HR + /HER2 − BC patients in both advanced and adjuvant settings. However, liver enzyme elevations are a common adverse event that often leads to dose changes or treatment discontinuation, highlighting the need for better management. While CTCAE classifications can overestimate severity, integrating DILI criteria may enable more precise and individualized care. Most ALT/AST elevations are mild and reversible, but early detection and monitoring remain critical.

Despite increased clinical use of CDK4/6i, guidance on interpreting and managing liver enzyme elevations is still limited across oncology and hepatology. This consensus aims to provide unified recommendations based on regulatory and real-world perspectives. Future work should validate hepatology classification systems within oncology, optimize algorithms to reduce interruptions, and further research the mechanisms and management of liver injury. Potential risk factors like race, fatty liver disease, or metabolic syndrome should not automatically prevent CDK4/6i use in eligible breast cancer patients.

## Data Availability

Survey data can be shared upon reasonable requests through a data sharing agreement.
